# Unraveling the Interplay of Dopamine, Carbon Monoxide, and Heme Oxygenase in Neuromodulation and Cognition

**DOI:** 10.1021/acschemneuro.3c00742

**Published:** 2024-01-12

**Authors:** Nicola Bauer, Dongning Liu, TanPhat Nguyen, Binghe Wang

**Affiliations:** Department of Chemistry and Center for Diagnostics and Therapeutics, Georgia State University, Atlanta, Georgia 30303, United States

**Keywords:** Dopamine, cognitive function, circadian rhythm, inflammation, heme oxygenase, carbon monoxide

## Abstract

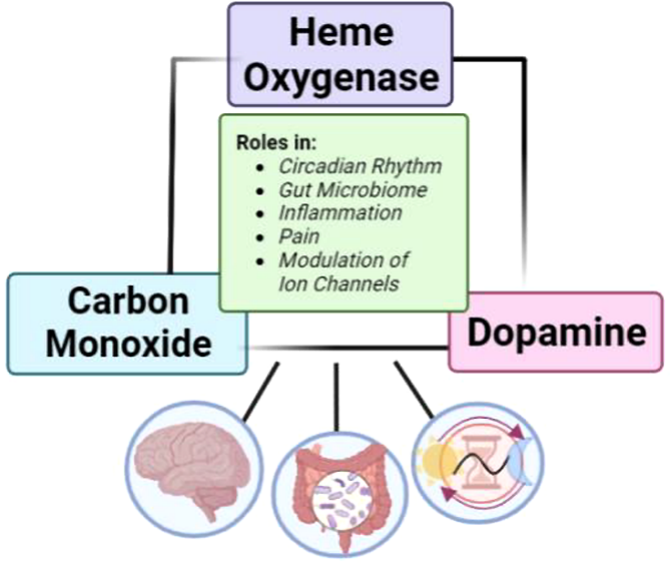

The dopaminergic system plays important roles in neuromodulation, including prominent roles in complex neurological functions such as cognition, reward, motivation, and memory. Understandably, the highly complex nature of such physiological functions means that their regulation is intertwined with other signaling pathways, as has been demonstrated by numerous studies. Contrary to its public perception of being poisonous at all concentrations, carbon monoxide (CO) is produced endogenously from heme degradation by heme oxygenase (HO) as part of the physiological process of red blood cell turnover. Physiological concentrations of CO can reach high micromolar ranges in the hemoglobin bound form. Low-dose CO has shown therapeutic effects in numerous animal models, including traumatic brain injury via engaging various hemoprotein targets. As such, the HO–CO axis has been shown to offer beneficial effects in organ protection, anti-inflammation, and neuroprotection, among many others. Further, a large number of publications have shown the interactions among CO, HO, and the dopaminergic system. In this review, we critically examine such experimental evidence in a holistic fashion and in the context of a possible dopamine–HO–CO signaling axis. We hope that this Perspective will stimulate additional investigations into the molecular connectivity related to this possible axis and open doors to the development of novel therapeutics that impact the dopaminergic system.

## 1 Introduction

The dopaminergic system plays important roles in neuromodulation including prominent roles in complex neurological functions such as cognition, reward, motivation, and memory. Understandably, the highly complex nature of the dopaminergic system necessitates the existence of intersection points with other signaling pathways fundamental to normal pathophysiological responses.^1−5^ For example, the dopaminergic system is implicated in injury,^6^ starvation and feeding,^7^ temperature changes,^8^ and cardiometabolism,^9^ among many others. As such, understanding how other signaling pathways intersect with the dopaminergic system is of great significance.

The HO–CO axis has been recognized as playing very important roles in various pathophysiological processes, including circadian clock regulation, inflammation, including neuroinflammation, and organ protection. Heme oxygenase (HO) has two isoforms, the inducible HO-1 and constitutive HO-2,^10^ and catalyzes the degradation of heme as part of red blood cell turnover, leading to endogenous production of carbon monoxide (CO, about 400 μmol/day) together with biliverdin and then bilirubin, as well as iron.^11,12^ Thus, significant CO production is part of a physiological process. This fact is often surprising to the wider biomedical community because of the widely known toxicity of CO at high levels and sometimes the deductive extrapolation of its toxicity at low levels, as well. It is important to note that under physiological conditions, CO can reach high micromolar concentrations in the hemoglobin-bound form, carboxyhemoglobin (COHb).^13^ At high concentrations, CO toxicity is an issue with induction of Parkinsonism, cognitive impairment, loss of consciousness, or even death. The key is the level of exposure, as Paracelsus, commonly credited as the founder of modern toxicology, correctly stated about 500 years ago: “The dose makes the poison.”^14^ Modern toxicologists widely accept the idea that the “dose–response relationship is a central concept in many biological disciplines but especially in pharmacology, toxicology and risk assessment.”^15^ Relevant to the discussion, even dopamine, naturally produced and needed for survival, is reported to be toxic at certain doses.^16^ Therefore, the beneficial pharmacological effects of the HO–CO axis lie within the boundary conditions of appropriate dosage much the same way as the use of other pharmaceuticals, such as insulin, blood thinners, anticancer drugs, and blood pressure medications, among many others.

**Figure 1:**
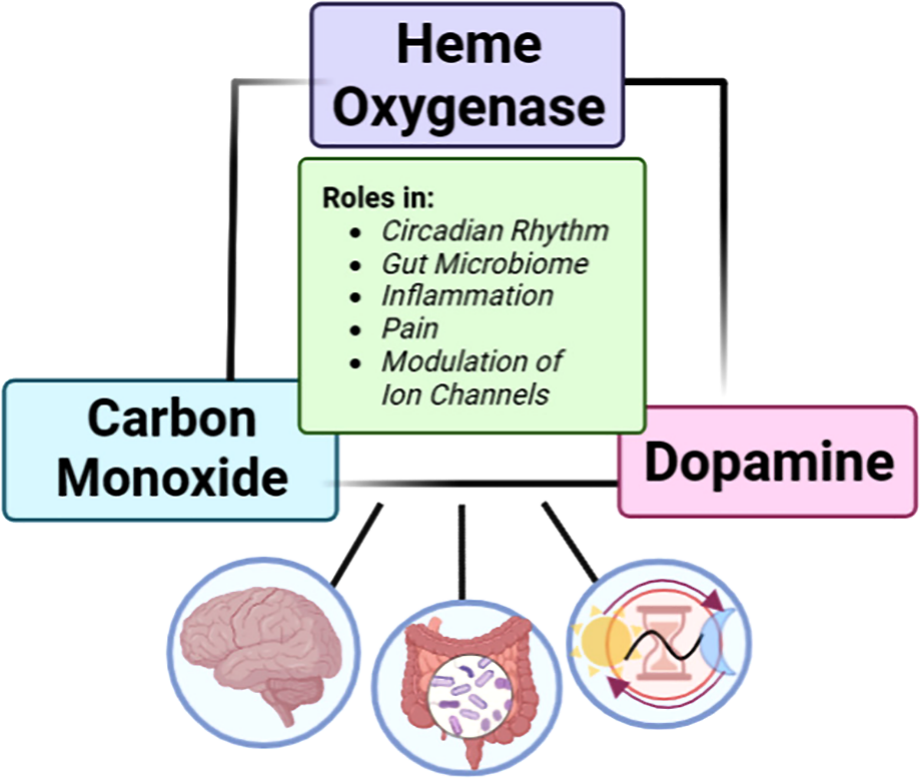
Links between dopamine and the HO–CO axis.

Directly relevant to the theme of this article, accumulating evidence directly indicates many intersecting points among dopamine, HO, and CO. For example, exogenous CO increases extracellular dopamine concentrations and inhibits dopamine reuptake, similar to many cognitive stimulants.^17,18^ Along the same line, low-dose CO has been reported as a neuroprotective agent in Parkinson’s disease, which is strongly linked to dopaminergic dysfunction.^19^ Further, dopamine has been reported to induce HO-1 and removal of endogenous CO alters dopamine release.^20−22^ Beyond exposure to systemic CO, the inducible isoform, HO-1, is expressed in the cerebellum and hippocampus and responds to oxidative stress and inflammatory stimuli.^23^ HO-1 has been reported to be induced in microglia and astrocytes by oxidative stimulus.^24^ On the other hand, HO-2 is constitutively expressed at high levels in glial and neuronal cells. It has been reported that the concentration of CO found in several types of neuronal cells ranges from 3 to 30 μM.^25^ The HO–CO axis has been widely reported to promote neuroprotection/neurogenesis and functional recovery in many neurorelated diseases.^26−28^ Along a similar line, CO has been reported to act as an atypical neuromodulator.^29^ Additional evidence within the literature points to a connection between HO-1, exogenous CO, and dopamine concentration in the brain.^17,18,20,21^ In this Perspective, we critically examine such experimental evidence in a holistic fashion and in the context of a possible dopamine–HO–CO signaling axis (Figure 1) with the hope that it will stimulate additional investigations into related molecular connectivity and allow for studies along this line to be put into a broader perspective. In discussing the existing experimental evidence, we note three aspects. First, it is important to note that all known molecular targets for CO in humans are hemoproteins in their ferrous state.^30^ For example, CO is known to reversibly bind to neuroglobin (Nb) with a high affinity (*K*_d_ as low as 0.2 nM), which is much lower than that of hemoglobin (Hb, *K*_d_ of 0.7 nM to 4.5 μM) or myoglobin (Mb, *K*_d_ of 29 nM). This means that CO transfer to Nb from Hb and Mb, the two largest reservoirs of CO in the body, is a thermodynamically favorable process. CO also binds to other hemoproteins with varying affinity including, but not limited to, neuronal PAS domain protein 2 (NPAS2, *K*_d_ of 1–21 μM), the Circadian Locomotor Output Cycles Kaput Protein (CLOCK, *K*_d_ of 100 μM), and cytochrome p450 (*K*_d_ of 1.4–10 μM). Second, this review is focused on results using CO gas and avoids the use of studies using known CO donors. This allows for a high degree of certainty of the active ingredient and avoids any complications from the “carrier” portion of the donor when discussing the complex nature of neurological studies. Third, dopamine has been implicated in many areas where CO function has been reported, including circadian rhythm, neuroinflammation, pain, gut microbiome regulation, and the modulation of ion channels. Though some evidence may be indirect, it is included in the discussion in order for us to present a complete landscape.

What the specific implications might be in the reciprocal interactions among dopamine, CO, and HO deserves much more work. The idea that CO and/or HO might have a role in dopamine-mediated functions, including cognitive functions, is an intriguing one. In this article, we have selected key examples to highlight the interplay among dopamine, HO, and CO and critically examine published experimental evidence in the context of molecular connectivity and the possible existence of a dopamine/HO/CO axis. However, we note that, to our knowledge, this is the first time such connectivity has been recognized in a holistic way. Therefore, we provide key examples from independent and individual studies to emphasize this holistic view of potentially a new signaling axis, with the hope to stimulate investigations into this intriguing area of research. Below we present these discussions.

## 2 Dopamine/HO/CO

To introduce a positive connection between dopamine and CO/HO, it is easiest to look at where functions of dopamine are generally understood. Dopamine is well-known in the scientific community to stimulate the brain and mediate cognitive effort.^31^ These broad functions of dopamine are becoming familiar to the general public due to the increasing prevalence of nootropics, like caffeine and prescription stimulants, which are often for patients with attention-deficit hyperactivity disorder (ADHD), a disorder riddled by cognitive and executive dysfunction.^32^ Many of these prescription stimulants are dopamine reuptake inhibitors (DRIs), which help improve cognitive and executive functions through increasing the extracellular concentrations of dopamine (DA) and increasing dopaminergic neurotransmission. Outside of ADHD, DRIs are sometimes prescribed to treat depression and other diseases with cognitive dysfunction, such as Parkinson’s disease.^33^ Although the idea of CO playing a role in cognitive stimulation is unexpected, the literature points to CO regulating dopamine through a mechanism similar to DRIs. Along the same line, connections between HO-1 induction by dopamine add another link to the proposed dopamine/HO/CO axis.

### 2.1 CO and Dopamine

CO has been reported to evoke dopamine release. In 1993, Hiramatsu and co-workers reported that rats treated with pure CO gas, in intervals of 30 s, 30 s, and 10 s, showed a 4-fold spike of DA concentration in the striatum within 15 min of treatment, which then returned to the baseline within 45 min.^17^ A different study by Taskiran and co-workers found that both male and female rats (although with more significant results seen in females) treated with pure CO, via bubbling into synaptomes for 1, 5, or 10 min, showed a maximal inhibition of DA reuptake at 10 min, where DA reuptake was inhibited by ∼78%.^18^ The CO-induced increase in extracellular DA concentration and inhibition of DA reuptake suggests CO could possibly follow a similar mechanism as a DRI. Although these two reports are based on the use of high concentrations of CO, Dreyer-Andersen and co-workers reported that low-dose CO enhanced survival and dopaminergic differentiation of human neural stem cells.^34^ In this study, cells treated with 25 ppm of CO at days 0 and 4 showed a significant increase in tyrosine hydroxylase expressing catecholaminergic neurons as well as an overall increase in the dopamine concentration. Since tyrosine hydroxylase is a dopamine producing enzyme, such results are congruent with each other. In a later publication, Ueno and co-workers reported that CO acts as a retrograde messenger to evoke noncanonical dopamine release in postsynaptic mushroom body neurons (MB) in *Drosophila*.^22^ In this study, it was found that suppression of HO activity, through both inhibition (chromium mesoporphyrin, CrMP) and HO knockdown (RU486, mifepristone), inhibited CO production as well as presynaptic DA release. The authors demonstrated that CO is endogenously generated in the lobes of MB neurons following coincident stimulation of the MBs using a fluorescent CO probe, COP-1. Then they showed that the direct application of CO, using CO-saturated saline, induced DA release from presynaptic terminals. Along the same line, they found that the addition of a CO scavenger, HemoCD, suppressed release. These results led to the conclusion that endogenously produced CO is required for DA release and that exogenous administration of CO at a concentration close to that of physiological range is likely sufficient to address pathophysiological deficiencies in CO production and evoke DA release. In summary, these results suggest that CO acts as a modulator of synaptic transmission and alters the concentration of important neurotransmitters such as dopamine. Furthermore, these studies point to the likelihood for CO to act as a cognitive stimulant with mechanisms similar to DRIs, prescription cognitive stimulants.

### 2.2 HO-1 and Dopamine

Interestingly, dopamine has been reported to be a potent inducer of HO-1.^20,21,35^ In 1999, Schmidt and co-workers reported that DA dose-dependently induces HO-1 mRNA in C6 glioma cells and astrocytes.^20^ It was found that micromolar (1–100 μM) and even submicromolar (100 nM) concentrations of DA upregulated the expression of HO-1. These results suggest that the DA released from neurons may be enough to trigger HO-1 in neighboring astrocytes. In agreement with Schmidt, Berger and co-workers reported a dose-dependent induction of HO-1 mRNA and antigen expression in HUVECs, cells that do not usually express HO-1.^21^ In this study, HO-1 became detectable with approximately 6 μM DA administration and showed a 4-fold induction when approximately 65 μM DA was administered. The kinetics of the HO-1 induction were studied. Treatment with 65 μM DA led to detectable levels of the HO-1 antigen at 8 h, and the levels peaked at 48 h. Under the same conditions, HO-1 mRNA was detectable at 2 h and peaked at about 6–12 h. These results agree with similar findings in a paper by Schmidt showing a peak in HO-1 mRNA level at 6–12 h after administration of 500 μM DA. Along the same line, Rider and co-workers found analogous results in SK-N-SH neuroblastoma cells, which showed no expression of HO-1 without induction.^35^ In comparison, as little as 1 μM DA resulted in detectable levels of HO-1, with no change in the constitutive HO-2 expression even at high concentrations of DA. It was also reported that dose-dependent HO-1 induction by DA was inversely correlated with cell survival; neuroblastoma cells treated with 10 μM DA showed an over 3-fold increase in HO-1 induction and 62% cell survival. These results suggest that DA is a potent inducer of HO-1 in various cell types. Taken together with the ability of CO to evoke DA production described in the previous section, such results suggest the likelihood of a HO-1/CO/DA axis.

## 3 The Circadian Clock

Circadian dysfunction is implicated in the development of many brain disorders including ADHD, autism, Alzheimer’s, and Parkinson’s disease. The circadian rhythm modulates the phases of critical functions such as sleep, metabolism, behavior, and cognitive function. Specific cognitive functions that have been reported to rely on the circadian rhythm include attention, working memory, cognitive conflicts, flexibility, and association.^36^ Interestingly enough, CO has been shown to interact with proteins in the circadian rhythm that act as molecular links to dopamine regulation in the circadian rhythm.

### 3.1 CO and the Circadian Rhythm

It has been reported that HO-2 generated CO and exogenous CO bind to the heme domain of NPAS2, which deactivates the transcriptional process of the transcription-translation feedback loop (TTFL). Dioum and co-workers reported that CO binds to the heme binding domain (HBD) of each NPAS2 monomer with *K*_d_ of 1–2 μM for PAS-A and 21 μM for PAS-B.^25^ The consequences of CO binding to the NPAS2 HBD was determined using an in vitro DNA binding assay, where at least 3 μM CO impairs DNA binding by holoNPAS2 through blocking NPAS2/BMAL1 heterodimerization.^25^ A different study found that the CLOCK PAS-A showed 65% sequence similarity to NPAS PAS-A and that CLOCK PAS-A similarly binds to CO and NO.^37^ These combined results suggest that one way for CO to regulate the circadian rhythm is by binding to the HBD of the key proteins in the primary loop of the TTFL. In terms of the secondary loop of the TTFL, Rev-erb is a protein that binds CO, where the *K*_d_ of CO to Fe^2+^-Rev-erbβ was determined to be 60 nM.^38,39^ CO’s role has also been studied through HO-1 inhibition or depletion of endogenous CO using hemoCD1, which is further described in a previous review.^30^ Although further mechanistic studies of CO and the circadian clock are needed to determine the exact role that CO plays, it is clear that both endogenous and exogenous CO play a role in circadian rhythm regulation through both TTFL loops. Further, the dopaminergic system intersects with regulation of the circadian rhythm.

### 3.2 Circadian Rhythm and Dopamine

The circadian rhythm has been linked to dopamine regulation through two key proteins as well. Inhibition of Rev-erbα has been reported to increase dopaminergic activity via inducing DA production.^40^ Genetic mutations in the CLOCK protein, ClockΔ19, have been associated with mania-like behavior through an increase in the midbrain dopaminergic activity.^40^ The circadian rhythm is reported to regulate dopamine. Along the same line, dopamine has been reported to modulate the circadian rhythm in the central nervous system (CNS) through a variety of factors.^41^ For example, disorders that are hallmarked by low dopamine, such as ADHD, have been reported to show disruption in levels of Bmal1 and Per2. CO’s role in mediating dopamine and the circadian rhythm adds to the credence of a possible dopamine/HO/CO axis.

## 4 Neuroinflammation and NF-κB

There have been many studies that have linked chronic pain with cognitive dysfunction including attention, learning, memory, information processing, general cognition, and executive function.^42^ Nonsteroidal anti-inflammatory drugs (NSAIDs) are commonly used drugs to treat inflammation and nociception.^42^ There has been conflicting reports on whether opioid, tricyclic antidepressants, or anticonvulsant treatment impairs cognitive function, but most reports agree that NSAIDs improve the cognitive function seen in chronic pain patients.^42^ Interestingly, through the inhibition of NF-κB, a proinflammatory transcription factor, CO could have similar mechanisms to NSAIDs.^43−45^

### 4.1 Dopamine and Neuroinflammation (NF-κB)

Dopamine receptors are present on various neuroimmune-related cells, including microglia and astrocytes.^46^ A dysfunctional dopaminergic system has been associated with neuroinflammation and linked to ADHD.^47^ Dopamine has been linked to anti-inflammation through NF-κB as well. Wu and co-workers reported that DA inhibited TLR2-induced NF-κB activation, subsequently suppressing inflammation.^48^ Along the same line, activation of D2R was reported to enhance the interaction of αB-crystallin with NF-κB, which blocked the DNA-binding activity of NF-κB.^46^ Interestingly, CO has been implicated in mediating pain through inhibition of NF-κB, as well as other anti-inflammatory effects involving activation of peroxisome proliferators activated receptor γ (PPARγ),^49,50^ hypoxia-inducible factor (HIF)-1α,^51^ mitogen-activated protein kinases (MAPK),^52^ and/or nuclear factor erythroid 2-related factor 2 (Nrf2).^53,54^

### 4.2 CO and Inflammatory Pain (NF-κB)

The main mechanism of action (MOA) of NSAIDs is through the inhibition of cyclooxygenases (COX-1,-2), blocking the production of inflammatory mediators such as prostaglandins, interleukin (IL)-2, IL-6, and tumor necrosis factor (TNF).^55^ Interestingly, induction of HO-1 has been reported to suppress LPS-induced COX-2 expression through the inhibition of NF-κB.^43,56^ Likewise, 250 ppm CO gas has been reported to interfere with LPS-TLR4 activation of NF-κB^44^ and inhibit NF-κB activation and DNA-binding of NF-κB after retinal I/R.^57^ Along the same line, the HO-1/biliverdin/CO axis has been implicated in the antinociceptive activity of selective COX-2 inhibitors.^45^ These results suggest that CO can mediate neuroinflammation and inflammatory-derived pain through inhibition of NF-κB. Although the exact mechanisms of CO on nociceptive and neuropathic pain need to be further evaluated, CO’s anti-inflammatory property prompts an interest in further studying the role of CO in mediating pain-related cognitive dysfunction and intersects with the function of DA on neuroinflammation.

## 5 Gut Microbiome

The human gut accommodates trillions of microorganisms, forming a microbial community called microbiome. Along the brain–gut–gut microbiota axis, the gut microbiome, the host brain, and the gut can bilaterally interact by means of signaling molecules. These complex processes appear vital to human health, as dysbiosis may impair cognitive functions, circadian rhythmicity, and human immune system.^58−63^ The axis between the brain and the gut microbiome has been well established in literature. The role of CO in the gut microbiome has been extensively reviewed by Hopper and co-workers.^64^

### 5.1 Gut Microbiome and Dopamine

The gut microbiome has been recently linked to many psychiatric and cognitive function disorders. Most microbiota found in the gut can produce different neurotransmitters such as dopamine and serotonin. More than 50% of DA is biosynthesized in the gastrointestinal (GI) tract,^65^ at a substantial rate of 12 nmol/min in mesenteric organs.^66^ The DA concentrations are regulated by the gut microbiome for signaling, and for modulating production of other neurotransmitters.^65^ Interestingly, many of the microbes that produce CO, including *E. coli*, *B. cereus*, and *L. brevis*, also produce neurotransmitters such as dopamine, serotonin, noradrenaline, γ-aminobutyric acid (GABA), and acetylcholine.^64^ This further implicates a role for CO as a neuromodulator in the gut microbiota.

### 5.2 Gut Microbiome and CO

The gut microbiome is known to produce, consume, and respond to CO, which suggests a CO-mediated multidirectional communication between the gut microbiome and the mammalian host.^64^ One way of production is through HO, where HO-1 is often inducible in the gastrointestinal (GI) tract under condition of stress or injury.^64,67^ HO-derived CO then targets several biomacromolecules based upon binding with high affinity in order to activate signaling pathways, namely, MAPK, ERK, JNK, JAK/STAT3.^64^ Aside from HO, there have been reports of natural products, carbohydrates, intestinal gas, and nutritional sources being broken down into CO via microbes present in the gut microbiome. The production of CO via breakdown of various substrates, such as vitamin B12,^68^ flavonoids,^69^ nutrient broth,^70^ glucose,^71^ and phenylpyruvate,^72^ provides a link between dietary nutrition and CO capacity.

## 6 Ion Channels

Ion channels play an important role in the CNS. They can be activated cyclically and release neurotransmitters that can provide unique physiological and pharmacological responses to different regions of the brain. There is some evidence that abnormalities in ion channels can increase the risk of cognitive disorders, such as Alzheimer’s,^73^ Parkinson’s,^74^ bipolar disorder and autism.^75^ Interestingly, one of the therapeutic mechanisms of CO is reported to be through the modulation of ion channels.^76,77^

### 6.1 K^+^ Channels and Dopamine

Inhibition of K^+^ channels has been shown to lead to an increase in dopamine levels.^78^ The G-protein-gated inwardly rectifying potassium (GIRK) channel is related to many cognitive disorders that are regulated by dopamine, such as epilepsy, Down’s syndrome, and Parkinson’s disease.^79,80^ There is also some evidence showing that ATP-sensitive K^+^ (K_ATP_) channels can inhibit dopamine release tonically by activation.^81^

### 6.2 ATP-Sensitive K^+^ (K_ATP_) Channels and CO

K_ATP_ channels are ubiquitously expressed in neurons located in the hippocampus and cortex of the brain. Animal studies have shown that the activation of endogenous K_ATP_ channels reduces cellular damage caused by cerebral ischemic stroke. This suggests that modulating K_ATP_ channels may in the future be used as part of combination therapies for stroke management.^82^ K_ATP_ channels are inward-rectifier potassium channels surrounded by four sulfonylurea receptors (SURs). One of the receptors, sulfonylurea receptor 2A (SUR2A), contains a heme-binding site of the K_ATP_ channel, the CXXHX16H motif, located in the cytoplasmic region. In 2018, Kapetanaki et al. found that CO can increase the channel activity by binding to heme, and in the absence of heme, CO’s activity on the K_ATP_ channel was lost.^83^ The binding affinity of CO with the heme was studied by a titration experiment using a CO saturated solution, and around 5 μM CO was able to titrate the heme complex. As a result, the dissociation constant for CO to the ferrous heme–SUR2A complex was determined to be around 0.6 ± 0.3 μM. These results demonstrate the ability for CO to regulate the K_ATP_ channel activity by binding to heme.

In summary, CO can bind with heme in the heme–HDM complex to regulate the functions of K_ATP_ channels, which are implicated in dopamine production. This suggests another link between CO and dopamine and the idea that both can regulate some ion channels that are highly relevant with the cognitive functions. CO has also been reported to regulate the channel functions of BK_Ca_ and K_v_ channels.^77^ Since these ion channels are also implicated in cognitive function, there is potential for CO to stimulate cognitive function through interacting with K^+^ ion channels.^84−86^

## 7 Conclusion

The specific implications in the reciprocal interactions among dopamine, CO, and HO deserve much more work. Although it is an unexpected idea that CO and/or HO might have a positive role in dopamine-mediated functions, including cognitive function, through many of its molecular targets, CO could work in cognitive function on a broad scale. A likely molecular link between CO and cognitive stimulation is seen through a possible dopamine/HO/CO axis, where CO has been reported to increase extracellular dopamine, inhibit dopamine reuptake, and increase dopaminergic activity. Along the same line, dopamine is known to be a potent HO-1 inducer, which ultimately leads to the increased production of CO. A dopamine/HO/CO axis and the role of CO in cognitive stimulation could be linked where roles of CO and dopamine are both seen, including the circadian rhythm, neuroinflammation, regulation of the gut microbiome, and modulation of ion channels. Along the same line, we pose questions on whether this could provide further linkage between reports on cigarette smoking (and thus elevated levels of CO ^87^) and the inverse association with cognitive function related disorders, such as Parkinson’s disease.^88^ Through proposing a possible dopamine/HO/CO axis, we hope to stimulate investigations into this intriguing area of research. Although no direct links in regulating this axis have been reported so far, two possible connections could be through protein kinase C (PKC) and neuronal hemoglobin (nHb). First, activation of PKC has been reported as a molecular mechanism of increased dopamine release.^89^ Along the same line, one mechanism of cognitive stimulating drug’s ability to increase dopamine efflux is through the modulation of PKC.^90^ Interestingly, CO has been implicated in the activation of PKC through HO, where the catalytic activity of HO-2 reportedly increases as a result of phosphorylation from PKC.^91^ Even further, CO has been directly implicated in activating PKC where CO-saturated medium reportedly increased cell migration to promote gastric wound healing through the activation of PKC.^92^ Second, Hb has been reported to be expressed in dopaminergic neurons and expression of nHb proteins has been reported to be colocalized in vivo in mouse dopaminergic neurons.^93,94^ It is suggested that nHb regulates the neurotransmission of dopamine cells.^95^ Since CO has a high affinity for hemoglobin, more research into this area would be interesting. Finally, we reiterate that the specific implications among dopamine, CO, and HO, along with any suggested possible regulatory factors, deserve much more work. We hope that this holistic view of a dopamine-HO–CO axis will stimulate future investigations.
